# Why don't we teach medical students to work in chaos?

**DOI:** 10.3389/fmed.2026.1701449

**Published:** 2026-02-10

**Authors:** Waseem Jerjes, See Chai Carol Chan, Marcin Klingbajl, Azeem Majeed

**Affiliations:** Department of Primary Care and Public Health, Faculty of Medicine, Imperial College London, London, United Kingdom

**Keywords:** adaptive expertise, cognitive load, complex adaptive systems, patient safety, simulation-based education

## Introduction

Health care rarely operates in a steady state. Clinicians and learners work in the face of fluctuating demand, workforce shortages, contradictory information, IT failures and time-critical escalation across emergency departments, acute wards, theaters and in the community. In OECD (Organisation for Economic Co-operation and Development) health systems, access constraints and workforce limitations continue to test performance in the pandemic rebound's aftermath, with persistent pressures evident in urgent and emergency care, elective waiting lists, and access to primary care (1).

In England, the CQC's (Care Quality Commission) most recent State of Care reports services being under sustained pressure across the board, with both access and quality being variable and recovery from the pandemic as incomplete (2). Recent winters have again shown that “surge conditions” are no longer exceptional, with performance against time-based access standards remaining under sustained strain (3, 4).

The implications for patient safety are profound. It is estimated that approximately 1 in 10 patients are harmed in health care and that unsafe care causes over 3 million deaths a year globally; in primary and outpatient care, 4 in 10 patients can be harmed, much of it being preventable (5). At the sharp end, the real-world work is fraught with interruptions and competing tasks. Observational studies demonstrated that interruptions are linked to greater danger and medication administration error severity and reduce the completion of tasks, reinforcing the necessity for explicit tactics for managing cognitive load under time pressured care (6, 7). These challenges require that newly graduated doctors be able to not just execute protocol under ordered systems, but also be capable of stabilizing care and making defensible choices when information is incomplete, inconsistent, or rapidly evolving.

UK standards already name this ambition. The General Medical Council (GMC) Outcomes for Graduates require new doctors to recognize and manage complexity and uncertainty, to escalate early when patients deteriorate, and to work effectively across settings and teams (8). Yet the conditions in which many trainees and trainers work impose substantial cognitive and emotional load. In the GMC National Training Survey 2024, over a fifth (21%) of trainees were measured as at high risk of burnout, and over half (52%) described their work as emotionally exhausting to a high or very high degree (9). The NHS Staff Survey 2024, drawing on responses from over 700,000 staff, paints a consistent picture of sustained operational strain across services (10).

Against this backdrop, multiple strands of evidence support a shift from teaching students to simply perform in order, to preparing them to perform when order breaks down. Safety science highlights the value of high reliability organizing and resilience—detecting, adapting to and recovering from variability (11); research on adaptive expertise advocates for preparing learners to flex beyond routine algorithms when faced with ill-structured problems (12); cognitive and team learning approaches (e.g., cognitive forcing strategies, structured debriefing, and situation awareness in teams) offer practical methods to build metacognition, shared mental models and reliable action under pressure (13–15).

This article is a conceptual synthesis rather than a systematic review, drawing on empirical strands from safety science, simulation, and adaptive expertise to justify why “chaos competence” should be taught explicitly. Direct evaluations of whole-curriculum “chaos competence” packages remain limited; therefore, the proposal is framed to be testable, with clear behavioral outcomes and assessment anchors that can be evaluated in future implementation studies.

### Why chaos competence is necessary?

Health care behaves less like a linear production process and more like a complex adaptive system in which disparate and interconnecting components—patients, clinicians, technologies, logistical and policy elements—produce non-linear, at times abrupt, demand and risk variations (16, 17). In this paper, complexity science provides the overarching lens; “chaos” is used more narrowly to describe the volatile edge of complex systems where small perturbations can trigger disproportionate change.

Within this complexity, periods of “chaos” can emerge when small disruptions—such as an IT failure, an unrostered shift, or a delayed ambulance handover—cascade across sites and rapidly reshape demand, risk, and priorities at the frontline. Such dynamics underline a central fact: the student doctors and newly graduated professionals will spend much of their early practice managing fluctuating queues, incomplete information, and time-critical decisions, rather than encountering tidy single problems.

At the bedside, interruptions and competing tasks are pervasive and consequential: studies link them with greater risk, more severe medication errors, and reduced task performance under constant distraction (6, 7). Acute stress narrows attention and reduces working memory, impairing performance unless learners can regulate arousal and externalize key information (18). Cognitive load theory explains why disorganization breeds error: where intrinsic and extraneous load exceed capacity, reasoning shortcut and fixation are more likely unless learners can reduce, sequence and slow down consciously (19). In short, disordered environments are routine and create fertile ground for error if graduates are inadequately prepared.

Complexity science and safety research offer an answer: high reliability organization places a strong emphasis on foresight, rapid detection and recovery in the face of difficulties, not simply rule following (11); naturalistic decision making details experts' behavior under time pressure by matching fluid cues against prior experience, knowing how far to slow down (16); and situation awareness offers a realistic cognitive template—perceive, understand, project—for sustaining a workable overview despite fluctuating circumstances (15, 17). Adaptive expertise in the science of the study of medical education fills out this answer by helping prepare learners to venture beyond routine algorithm when difficulties are ill structured, but in a manner which avoids abdicating standards or responsibility (12).

Individually, these threads warrant explicit teaching toward chaos. Should our curriculum condition students only for predictable, single-problem scenarios, then safety is left to chance and personal heroics when systems behave, as they too often do, non-linearly. Chaos competence is therefore not an optional extra, but a key professional skill grounded in the systems we already have and the standards we already demand (1–5, 8–12, 15–18).

### What to teach?

Chaos competence is best conceived as a deliberately taught constellation of micro skills that help learners notice early, prioritize effectively, act safely and re-plan as conditions shift. The cognitive backbone is naturalistic decision making: under time pressure, clinicians often match evolving cues to patterns from prior experience, yet must recognize when to slow down and switch to analytic reasoning to avoid fixation (13, 16, 17). Situational awareness—perceiving key cues, comprehending their meaning and projecting what may happen next—provides a practical scaffold for prioritization and re-prioritization as new information arrives (17–20). Because interruptions and partial data are common, learners need explicit tactics for cognitive load management (externalizing information, chunking tasks, short “reset” pauses) to prevent overload and error (6, 21, 22). Cognitive aids such as crisis checklists can safely offload memory while preserving clinical judgement when drills and debriefs have normalized their use in practice (14, 23).

Capability in chaotic settings also depends on regulating one's own emotions and responses. Severe stress narrows the bandwidth of attention and impairs working memory. When unprepared, performance tends to fail at precisely the moments of greatest risk (18, 24–27). Education then must incorporate stress inoculation under titrated arousal, rapid in-the-moment regulation procedures (breathing drills, cognitive reframing) and formal debriefing linking emotional state, state of mind and quality of decision-making in order to convert difficult episodes to metacognitive learning (14, 18, 22–30). Students need to be urged to recognize overload warning signs in advance, initiate time outs and access support before thresholds are exceeded. It is important to frame professional identity in terms of safe escalation rather than lone heroics (10).

Most chaotic episodes are team-based challenges. Evidence concludes that team training—specifying roles, creating shared mental models and practicing mutual monitoring— can optimize processes and, in chosen environments, outcomes (19, 20). Communication requires a common spine: standardized handovers such as SBAR and routine closed-loop exchanges reduce omission and ensures plans are heard amidst noise (21–23). Non-technical skill models (e.g., NOTSS and ANTS) provide shared language and observable behaviors for feedback regarding situation awareness, decision making, leadership and communication (24–26). Because real clinical work often crosses boundaries, interprofessional rehearsal with pharmacy, nursing, and paramedicine teams should become standard, allowing students to practice assertive followership, respectful challenge, and cross-team escalation (31–41).

Finally, task and system fluency translate individual competence into dependable action and map closely to management reasoning—prioritization, trade-offs, monitoring, and re-planning beyond the diagnosis in time-pressured care (42), where “good” decisions are often context-dependent and shaped by resources, constraints and patient preferences. Students must practice interruption management (recognize, safely park, continue), transparent planning (articulate one line objective, key risks, next action), explicit safety netting and prompt risk stratification, with previously rehearsed answers to predictable failure modes, such as IT failure or abrupt deterioration (6, 24, 27). Clarity about ethics and the law is built in, rather than optional. Education must include triage ethics (equity, proportion, transparency), reasoning and documentation support for allocation decisions, and duty of candor communication in the face of doubt, all rooted in professional standards (8, 29, 30). Feedback must make clear that action in doubt is prized and coachable. Entrustable Professional Activities (EPAs) can specify the expectations for initial assessment/triage, handling a deteriorating patient and managing parallel tasks, with entrustment decisions based on direct observation of prioritization, escalation and safe switching of tasks, then supplemented with feedback and debriefing (14, 23, 32–36).

In short, the skill set combines rapid pattern recognition with debiasing, emotional regulation with team knowledge, and operational flow control with values-based transparency. Deliberately taught and tested, these skills enable graduate students to maintain safety for the patient under circumstances where the order fails, not just when the order is maintained (6, 8, 13, 14, 16–18, 21–27, 29–31, 37). The proposed domains, methods, and assessment anchors are mapped in Table 1.

**Table 1:** Curriculum map for “chaos competence” in undergraduate medical education.

**Domain**	**Learning outcomes (behavioral)**	**Embed within existing teaching (suggested methods)**	**Assessment approach**	**Observable indicators**
Cognitive and diagnostic	Maintains situational awareness; prioritizes dynamically; uses recognition-primed decisions but switches to analytic reasoning when needed; avoids fixation; documents rationale under uncertainty.	*In-situ* simulation with conflicting cues; tabletop decision exercises; micro-drills that force re-prioritization; coached use of cognitive forcing strategies; cognitive-load management tactics; crisis checklists for memory offload.	Cross-cutting EPA (“Stabilize, prioritize and re-plan care in volatile conditions”) embedded within existing EPAs (initial assessment/triage; deterioration; parallel task coordination); dynamic OSCE with mid-station change (interruptions, new data, escalation); workplace-based assessment with explicit “chaos triggers.”	States one-line goal and top risks; updates plan aloud when cues change; names uncertainty and safety nets; appropriate checklist deployment without tunnel vision.
Personal and affective	Regulates arousal under pressure; recognizes overload and calls time-out; seeks help early; links emotion to attention and decision quality; reflects and adapts after events.	Stress-inoculation practice with titrated complexity; brief regulation drills during scenarios (breathing, cognitive reframing); high-quality debriefing to consolidate metacognition.	Portfolio reflections with faculty sign-off; observed time-outs and help-seeking in simulation and clinical settings; rating of regulation behaviors during debrief.	Uses reset pauses appropriately; verbalizes limits; switches tasks or escalates before threshold breach; articulates “do differently next time” commitments in debrief.
Team and communication	Builds rapid shared mental models; clarifies roles; uses closed-loop communication and graded assertiveness; escalates across boundaries; hands over with a structured format.	Interprofessional *in-situ* simulation (medicine, nursing, pharmacy, paramedicine); team training; rehearsal of SBAR/I-PASS handover; brief-execute-debrief cycles.	Group OSCE sampling team cognition; EPA for team leadership/followership; mini-CEX focused on handovers; feedback using NOTSS and ANTS language for non-technical skills.	Clear task allocation and read-backs; concise shared summary (“situation, risks, next step”); timely, respectful challenge; effective cross-team escalation.
Task and systems	Manages interruptions safely; controls work-flow during surges; uses cognitive aids; anticipates deterioration; applies safety-netting and documents trajectory; rehearses downtime/escalation pathways.	Interruption drills and “clinic blackout” tabletops; short single-tasking bursts for critical steps; use of crisis checklists; scheduled time-outs; rapid risk-stratification routines; *in-situ* walk-throughs to surface latent threats.	DOPS/mini-CEX with chaos triggers; EPA for coordinating parallel tasks; dynamic OSCE stations testing re-planning and flow control.	One-line plan visible; prioritization of queue with rationale; one-touch rule for simple tasks; early call for help with clear threshold; documented reasoning and safety nets.
Ethical and legal	Applies triage ethics (fairness, proportionality, transparency) and recognizes bias risk under pressure; documents reasoning and escalation clearly; aligns actions with professional standards and inclusive communication.	Case-based debates and moral case deliberation embedded in scenarios; write-ups of resource-allocation decisions; rehearsal of candor conversations during debrief.	Case write-ups and viva focused on reasoning transparency; workplace-based assessments commenting on fairness and documentation quality; portfolio evidence mapped to national outcomes.	Explicit statement of ethical rationale; traceable documentation of trade-offs; timely disclosure and escalation; consistent linkage to organizational and professional guidance.

### How to teach and assess it?

Teaching for chaos should follow a developmental principle: begin with stable prototypes, then introduce complexity deliberately rather than leaving it to chance. Early curricula should prioritize tidy prototypes and core routines so learners can anchor safe defaults. Complexity can then be introduced in a staged way: first through controlled variability (extra comorbidity, contradictory cues, interruptions), then through dynamic team scenarios that require re-planning and escalation. Only once foundational patterns are stable should training emphasize high-volatility conditions where small perturbations cascade and priorities must shift rapidly. This sequencing ensures that “complexity by design” supports progression rather than overwhelming novices.

The most defensible architecture blends short, high-frequency practice to stabilize core moves with simulation that integrates cues and decisions, followed by supervised clinical consolidation. Systematic reviews and meta-analyses show that well-designed simulations with clear objectives, feedback and deliberate practice improves performance and can outperform traditional exposure for targeted skills, especially when challenge is escalated thoughtfully (32, 33, 38). These gains depend on psychological safety from the outset; robust pre-briefs and skilled debriefing can transform stress into learning, whereas poorly conducted sessions will add stress without skill (14, 36, 37).

*In situ* simulation is the best available proxy for true clinical volatility since it occurs in the real workplace among the real people, workflows and constraints. It reliably brings latent safety threats to the surface, tests cross-boundary coordination and lets educators to design the interruptions, missing data and equipment malfunctions that characterize episodes of chaos— and then examine how teams identify problems, adapt, and recover (39). Where manikins or gated time are a luxury, tabletop walk throughs and decision drills can simulate decision density at negligible expense, while micro drills (90 second scans, scripted “bleeps,” quick “time out” resets) compress the feedback loop. Stress inoculation helps educators calibrate arousal and coach regulation in real time, while cognitive load strategies support information externalization, task chunking, and short resets to prevent overload during interruptions (6, 7, 18, 27).

Cognitive aids and structured communication should be taught and practiced to fluency, so that checklists and structured handovers reduce omission without impairing judgement in noisy settings (21, 22, 24, 34, 35). Because chaos is interprofessional, scenarios should routinely include nursing, pharmacy and paramedicine teams, using shared non-technical skill language (NOTSS, ANTS) for feedback across disciplines (19, 20, 25, 26, 31).

Assessment must signal that action under uncertainty counts. EPAs offer a defensible framework: initial assessment and triage, managing deterioration and coordinating parallel tasks can be defined with observable anchors for prioritization, escalation, re-planning and safe task switching; entrustment decisions are then grounded in direct observation and coached over time (23, 40). One cross-cutting “Chaos competence” EPA could be framed as: “Stabilize, prioritize and re-plan care in volatile conditions,” with entrustment anchored to maintaining a one-line goal, updating the plan as cues change, escalating appropriately, and documenting reasoning transparently.

OSCEs (Objective Structured Clinical Examination) remain useful if they reflect reality: stations can incorporate mid-scenario changes—such as contradictory data, interruptions, or equipment failure—so assessors can observe detection, adaptation, and communication under pressure rather than static rule application (27, 41). Workplace-Based Assessment (WPBAs) should include explicit “chaos triggers” and narrative feedback on situation awareness, interruption management and escalation clarity, with reasonable adjustments to ensure fairness, and mapping to GMC Outcomes for Graduates to align expectations with what graduated doctors must do in complex, uncertain settings (8).

In practice, starting where variability is already high—emergency departments, acute medical units, theater lists, and urgent primary care—allows programmes to iterate quickly and evaluate impact in real workflows (32, 37). To reflect the meta-analytic signal that well-designed simulation translates into practice beyond the skills lab, evaluation should move beyond satisfaction scores to behaviors in the clinical environment and, where feasible, process or safety proxies (33, 38–42).

## Discussion

The case for teaching chaos competence is not a license for glorifying chaos nor a justification for tolerating unsafe staffing. It is a pragmatic response to the reality that health systems tend inherently toward unpredictability. Patient safety statistics show that harm remains common and too often avoidable, particularly where care is fragmented or delivered under time pressure (5). In this context, national standards already expect newly graduate doctors to respond responsibly in complex, ambiguous settings, to recognize and escalate deterioration, and to contribute across settings and teams (8). Questionnaires of trainees and workers validate that real-time environments are difficult, especially with high volumes of work and elevated risk of burnout (9, 10). In short, the current state already exposes students to environments of chaos; the key question is whether we expose them purposefully and prepare them to endure such conditions safely.

The educational shift is from curricula designed primarily for order toward curricula that incorporate adaptive capacity. Safety science and complexity research suggest that safe systems do not succeed by eliminating variation, but by recognizing it early, adapting, and recovering. “Work as done” systematically differs from “work as imagined,” and high reliability organizations place greater emphasis on learning and anticipating than in rule-based compliance (11). At the cognitive level, clinicians are making countless decisions under interruption and limited information, where the risk of error is high and performance deteriorates without explicit strategies to manage cognitive load (6, 7, 27). Naturalistic decision making and situation awareness explain how experts respond quickly under pressure and, more significantly, know when to slow down and cross-check (16, 17); adaptive expertise reframes competence as the ability to step beyond routine scripts where problems are ill-defined, while still maintaining responsibility and standards (12).

Internationally, this complements established system-aware educational frameworks that operationalise adaptive expertise, including the American Medical Association–supported Master Adaptive Learner model and the ACGME competencies of Systems-Based Practice and Practice-Based Learning and Improvement, which similarly foreground learning, adaptation, and improvement within real care systems (43).

These threads converge in a teachable repertoire—recognize, prioritize, act, re-plan—that mirrors how clinicians maintain patient safety while working in constantly shifting environments.

Implementation must be feasible within contested curriculum space, so “chaos competence” should be designed as an integration strategy rather than an additive curriculum layer (32, 33, 38). Instead of creating new blocks of teaching, programmes can embed chaos triggers into existing patient safety teaching, simulation, acute care placements and primary care attachments by modifying scenarios, handovers and assessment prompts (14, 36, 37). A minimum viable core could include a shared language for situational awareness and re-planning, two cognitive load strategies, one team communication spine, and a cross-cutting EPA that is used consistently in OSCEs and workplace assessment; this keeps the approach practical while avoiding additional curriculum blocks (39). Critically, any additions should be balanced by substitution: retiring lower-yield didactic sessions that duplicate content already covered and repurposing existing stations rather than expanding assessment time (19, 20, 25, 26, 31).

Because both patient harm and educational opportunity are unequally distributed, teaching must explicitly address how bias and structural disadvantage shape decision-making under pressure. Assessment design should support inclusion by using transparent anchors, reasonable adjustments where needed, and by avoiding any assumption that visible distress is a proxy for competence.

The practical next step is to define a minimal national core—capabilities, teaching designs and assessment anchors—mapped to GMC Outcomes for Graduates, so that medical schools can iterate rather than invent *de novo* (8, 23). Evaluation should extend beyond satisfaction scores to observed behavior in clinical practice to reflect the meta-analytic evidence that well-designed simulation translates into practice (33, 38). Framed this way, “working in chaos” becomes a core professional capability—one we deliberately teach and assess—so that patient safety does not rely on luck or heroics when order breaks down.
